# Syndromic surveillance for influenza in two hospital emergency departments. Relationships between ICD-10 codes and notified cases, before and during a pandemic

**DOI:** 10.1186/1471-2458-11-338

**Published:** 2011-05-18

**Authors:** Karen Moore, James Black, Stacey Rowe, Lucinda Franklin

**Affiliations:** 1 Monash University, Melbourne, Victoria, Australia; 2 Nossal Institute for Global Health, The University of Melbourne, Victoria, Australia; 3 Department of Health, Melbourne, Victoria, Australia

## Abstract

**Background:**

Interest in the use of emergency department (ED) data by syndromic surveillance systems to detect influenza outbreaks has been growing. Evaluations of these systems generally focus on events during influenza seasons. The aims of this study were to identify which emergency department disease codes best correlated with confirmed influenza cases and to determine if these same codes would be useful in the non-influenza season. The 2009 influenza pandemic in Victoria, Australia, provided further opportunity to examine the performance of the syndromic surveillance system during this event.

**Methods:**

We undertook a retrospective analysis of data from the Victorian Department of Health's pilot syndromic surveillance programme, 'SynSurv'. SynSurv automatically captures patient information as it is entered by ED staff. This information includes patient demographics, their presenting symptoms and a preliminary diagnosis using ICD-10 coding. To determine which codes were best correlated with influenza notifications, weekly counts for each of the ICD-10 diagnosis codes ever used in the dataset were calculated and compared with the corresponding weekly count of confirmed influenza cases. Correlations between these codes and confirmed influenza cases in the non-influenza season were then undertaken. The data covered the period from July 2001 until August 2009 and included the 2009 influenza pandemic.

**Results:**

There was a marked increase in weekly counts of both laboratory-confirmed influenza cases and relevant ICD-10 codes during the influenza pandemic period. The increase in laboratory confirmed cases was more than four times greater than the previous highest number reported, in 2007, even though the influenza-like-illness activity in the community was considered comparable to 2003 and 2007. We found five ICD-10 codes to be moderately and significantly correlated with influenza cases. None of these codes was correlated with laboratory confirmed influenza notifications outside the influenza season, at least in part because of the small number of influenza cases notified during that period.

**Conclusions:**

This study suggests that the choice of codes made by ED staff to record a case of influenza-like illness is influenced by their perceptions of how much influenza is circulating at the time. The ability of syndromic surveillance to detect outbreaks early may be impeded because case diagnosis is influenced by what ED staff believes to be occurring in the community.

## Background

The cost of seasonal influenza to communities worldwide is considerable [1]. In Australia the full extent of morbidity and mortality attributable to seasonal influenza is not known, although it is estimated to be responsible for around 2,800 deaths each year [2]. Surveillance undertaken by health departments plays an important role in the management of seasonal influenza. It provides information on the virus strain type and the level of virus circulating in the community, both of which can assist in evaluating the effectiveness of the current season's vaccine formulation. In the Australian state of Victoria the principle methods for influenza surveillance include passive and sentinel surveillance notifications of laboratory confirmed cases [3]. Both these methods incur substantial time delays between a case being first observed and notification to the health department while waiting for the results of laboratory testing. In the past these traditional surveillance methods have performed adequately in assisting in the control and prevention of outbreaks, but with mass global transport and a large, very mobile population, such traditional systems have limited ability to provide the rapid response required to avert modern epidemics [4].

One method that may assist in the early detection of disease outbreaks is syndromic surveillance. The two key components of syndromic surveillance that facilitate the early identification of outbreaks are the use of the clinical symptoms associated with a disease, rather than laboratory confirmed diagnoses, and the rapid and automatic collection and analysis of electronic data to generate alerts. These systems aim to identify increases, above normal background levels, in the incidence of particular disease syndromes. The trade off for this early warning, however, is lower specificity of the data collected [5].

A commonly used source of information for syndromic surveillance systems is data collected in emergency departments (ED) [6]. Emergency departments code clinical diagnoses of people presenting using standardized coding methods. The International Classification of Diseases, tenth revision (ICD-10) contains a number of respiratory diagnosis codes that when used alone or in combination should provide an accurate indication of influenza cases. The specificity of the data collected can be improved by ensuring that the ICD-10 codes used to define a specific syndrome are good indictors of the disease in question.

Studies investigating the effectiveness of syndromic surveillance to detect influenza outbreaks have generally focused on events during typical influenza seasons [7-9]. In contrast, this study also examined the effectiveness of syndromic surveillance outside the typical influenza season. The 2009 influenza pandemic in Victoria provided an added opportunity to examine the performance of a pilot syndromic surveillance system during this event.

This study had three objectives. The first was to determine which ICD-10 diagnosis codes used by clinicians in the emergency departments were best correlated with laboratory confirmed influenza cases notified to the Department of Health. The second was to determine if the codes found to be best correlated with total counts of laboratory confirmed notified cases were also correlated with influenza case counts during the non-influenza season. The third was to examine the effect of the 2009 influenza pandemic on the ED surveillance data.

## Methods

We undertook a retrospective analysis of data from the Victorian Department of Health's ED syndromic surveillance pilot programme, 'SynSurv' and from the Victorian Department of Health Notifiable Infectious Diseases Surveillance (NIDS) system.

### Ethical Approval

The Monash University Human Research Ethics Committee granted exemption from ethical review because the data was from existing surveillance registries and irreversibly de-identified.

### Data Sources

On 1 July 2005 the Victorian Department of Health implemented a pilot syndromic surveillance program called SynSurv at two major hospitals in Melbourne. All Victorian hospital emergency departments routinely collect patient information, known as the Victorian Emergency Minimum Dataset (VEMD), though not in real time, as part of the Victorian Government's reporting requirements. The SynSurv system automatically captures a subset of these data as they are entered by ED staff. Further details of SynSurv can be found in Newell and Black [10]. The data include demographic (age, gender and postcode of residence), administrative and clinical details on each patient who attends. The clinical data include the presenting symptoms (as free text) and the allocation of a preliminary diagnosis using ICD-10 coding. Up to three ICD-10 codes can be entered by the ED staff. This information is transmitted automatically and rapidly to the Department of Health as an encrypted package where the SynSurv application processes new data every five minutes [10].

Laboratory confirmed influenza became a notifiable disease in Victoria in 2001. All laboratory confirmed influenza cases notified to the department are captured in NIDS. We examined all cases notified between 6 July 2001 and 7 August 2009. The data include notification date, age, gender and postcode of residence, as well as details of the influenza strain type. For the analysis the corresponding data from the SynSurv database were used. Data from 1 July 2001, prior to SynSurv being operational, were imported from the VEMD dataset to supplement it. Fifteen weeks of data during the period that SynSurv had been on-line were found to be incomplete. These incomplete weeks were also supplemented with data from the VEMD.

### ICD-10 codes associated with influenza

Three analyses were conducted to investigate which ICD-10 codes best correlated with laboratory confirmed influenza notifications. The first was on the entire NIDS and SynSurv datasets. The second was on a subset of the dataset restricted by postcode. The two hospitals that provided the syndromic surveillance data are tertiary hospitals and provide specialist services to patients throughout the state. As influenza presentations to these hospitals would be expected to come mostly from the local area both the data from SynSurv and from the NIDS dataset were restricted to include records with postcodes that were within 10 kilometres of each hospital.

The third analysis excluded the 2009 influenza season as this was characterized by the H1N1 pandemic.

No 'a priori' decisions were made as to which ICD-10 codes should be analysed. Instead weekly counts for each of the ICD-10 diagnosis codes ever used in the dataset were calculated, although diagnoses with total counts of 200 or less were not included in the final analysis. Week of ED visit was used for the SynSurv time series and week of disease notification was used for the NIDS time series.

To identify which codes were associated with influenza, pair-wise correlations were calculated for each diagnosis time series, comparing the weekly event count in each series with the corresponding weekly count from the NIDS database. The calculation of correlation coefficients was repeated for the datasets after they were restricted by postcode and exclusion of the 2009 influenza season. The ICD-10 codes were ranked in decreasing order of strength of correlation.

As previous studies had found that syndromic surveillance could detect outbreaks before traditional surveillance methods [11,12] an analysis was conducted using one, two and three week time lags. Syndromic surveillance data were compared to NIDS data that had been collected one, two or three weeks later. A substantial increase in the strength of the correlation would suggest that syndromic surveillance data was identifying potential influenza cases earlier than the NIDS surveillance data. As the data was not normally distributed Spearman's Rank Correlation method was used.

### Analysis of ICD-10 codes by Influenza season

To identify which ICD-10 codes were best correlated with laboratory confirmed influenza cases notified to the department in the non-influenza season, the analyses above were repeated using data restricted to either the influenza season or the non-influenza season.

To determine the start and end of each year's influenza season, the mean weekly count of confirmed influenza cases (from the NIDS database) was first calculated for the clearly non-season periods before week 18 and after week 48 of each year. Each year's influenza season was defined as starting in the week beginning a sustained increase in laboratory confirmed cases to at least the out-of-season mean plus one standard deviation. The end of the influenza season was defined as the week beginning a sustained decrease back to the average count of out-of-season cases.

### Effect of 2009 influenza pandemic on ED surveillance data

To examine the effect of the 2009 pandemic on ED surveillance data we repeated the first analysis, to identify which ICD-10 codes were best correlated with notified cases of influenza, but excluded the 2009 influenza season from the data.

### Inter-hospital consistency

Correlations between the weekly counts for each ICD-10 code and counts of laboratory confirmed cases in the NIDS database were calculated for each hospital individually to compare the ICD-10 coding patterns between the two hospitals. The correlation patterns were similar between the two hospitals; both identifying the same top four ICD-10 codes (data not shown).

## Results

There were a total of 735,452 presentations to the two emergency departments during the study period captured in the SynSurv database. A diagnosis code was not included in 155,188 records. The ED staff could enter up to three diagnosis codes for each patient, so the total number of records used in the analysis for this period was 596,468. In total 644 different ICD-10 diagnosis codes were used by ED staff.

The NIDS database contained 12,050 notified influenza cases. There were 11,649 notified cases during the influenza seasons and a total of 401 notified cases during the non-influenza seasons.

### Postcode restriction

After restricting for postcodes within a 10 km radius of each hospital, there were 434,160 SynSurv records with a diagnostic code entered. This is approximately 73% of the total number of records available for analysis from the combined hospitals.

After restricting the NIDS database by postcode there were 4,391 notified influenza cases; 4,190 during the influenza season and 201 in the non-influenza season.

### ICD-10 codes correlated with laboratory confirmed influenza cases

Using the data for the whole state and all years, five ICD-10 codes were identified that had positive correlation coefficients greater than 0.3 and p-values less than 0.05 (Table 1). All were plausible surrogates for influenza. Analysis of the lagged time series data found no substantial increases in the strength of the correlations after adjusting for one, two or three week lags (Table 1).

**Table 1:** Correlation coefficients calculated between ICD-10 coded cases and NIDS cases with and without lag periods: all Victorian data, all seasons, 2001-2009

ICD-10 Code	Description	No lag*	1 week lag*	2 week lag*	3 week lag*
J11	Influenza virus not identified	0.481	0.465	0.493	0.438

J06	Acute upper respiratory infection multiple and unspecified sites	0.473	0.488	0.486	0.458

J22	Unspecified acute lower respiratory infection	0.433	0.429	0.447	0.380

B34	Viral infection, unspecified site	0.398	0.436	0.393	0.358

J18	Pneumonia organism unspecified	0.302	0.310	0.306	0.316

* p < 0.0001

**Figure 1:**
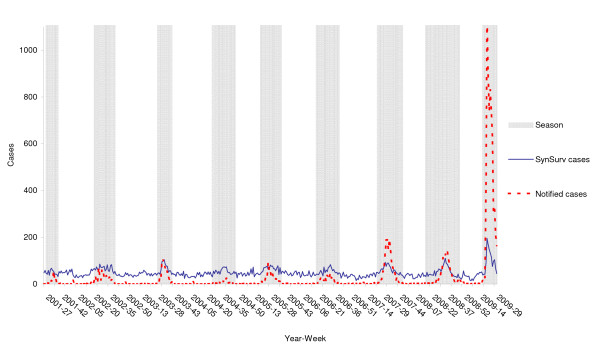
**Graph of the 'best' five ICD-10 correlations and confirmed influenza cases from the NIDS dataset**. Data for all of Victoria and all seasons 2001-2009.

These five ICD-10 codes were combined and analysed (Figure 1). The combined correlation was slightly higher than the individual correlations (Table 2).

**Table 2:** 'Best' ICD-10 diagnoses and correlations with NIDS cases, for all seasons, 2001-2009

ICD-10 code	Description	Full dataset		Postcode restricted	
		**Correlation**	**p**	**Correlation**	**p**

J11	Influenza virus not identified	0.481	< 0.0001	0.419	< 0.0001

J06	Acute upper respiratory infection multiple and unspecified sites	0.473	< 0.0001	0.452	< 0.0001

J22	Unspecified acute lower respiratory infection	0.433	< 0.0001	0.391	< 0.0001

B34	Viral infection, unspecified site	0.398	< 0.0001	0.405	< 0.0001

J18	Pneumonia organism unspecified	0.302	< 0.0001	0.250	< 0.0001

'Best' codes combined (r*_s _*>0.3)		0.557	< 0.0001	0.560	< 0.0001

Restricting the data by postcode resulted in slightly weaker correlations. Four of the ICD-10 codes identified above, all plausible surrogates for influenza like illness, had positive correlation coefficients greater than 0.3 and p values less than 0.05 (Table 2).

### Analysis excluding the 2009 influenza season

The influenza pandemic in 2009 resulted in more influenza notifications for that season than the total for all previous seasons in the dataset. There were 6,481 influenza notifications during the 2009 influenza season compared to the next largest number of notifications which was 1,533 during the 2007 influenza season. Analysis excluding the 2009 influenza season identified the same five ICD-10 codes but with slightly lower correlations. There were no appreciable increases in the strength of the correlations after including one, two or three week lags (Table 3).

**Table 3:** Correlation coefficients calculated between ICD-10 coded cases and NIDS cases, with and without lag periods: All Victorian data, excluding the 2009 influenza season

ICD-10 Code	Description	No lag*	1 week lag*	2 week lag*	3 week lag*
J11	Influenza virus not identified	0.433	0.417	0.456	0.404

J06	Acute upper respiratory infection multiple and unspecified sites	0.433	0.451	0.452	0.429

J22	Unspecified acute lower respiratory infection	0.424	0.417	0.435	0.367

B34	Viral infection, unspecified site	0.356	0.401	0.364	0.332

J18	Pneumonia organism unspecified	0.301	0.308	0.308	0.324

*p < 0.0001

The analysis was performed on both the whole-state and postcode restricted data. Postcode restriction resulted in small changes in the strength of the correlations and found four ICD-10 codes with correlations greater than 0.3 and p-values less than 0.05 (Table 4).

**Table 4:** Top correlations calculated between ICD-10 coded cases and NIDS cases after excluding the 2009 influenza season

ICD-10 Code	Description	All data		Postcode restricted	
		**Correlation**	**p**	**Correlation**	**p**

J11	Influenza virus not identified	0.433	< 0.0001	0.368	< 0.0001

J06	Acute upper respiratory infection multiple and unspecified sites	0.433	< 0.0001	0.409	< 0.0001

J22	Unspecified acute lower respiratory infection	0.424	< 0.0001	0.387	< 0.0001

B34	Viral infection, unspecified site	0.356	< 0.0001	0.365	< 0.0001

J18	Pneumonia organism unspecified	0.304	< 0.0001	0.262	< 0.0001

'Best' codes combined (r*_s _*>0.3)		0.529	< 0.0001	0.526	< 0.0001

### Comparison of influenza season and non-influenza season

The influenza seasons were from week 29 to week 40 in 2001; 22 to 41 in 2002; 29 to 42 in 2003; 27 to 48 in 2004; 20 to 39 in 2005; 20 to 41 in 2006; 25 to 48 in 2007; 18 to 49 in 2008; and from week 18 to the end of the study period in 2009.

During the influenza seasons there were four plausible ICD-10 codes with correlation coefficients greater than 0.3 and p-values < 0.05; B34, J11, J06, and J22 (Table 5). After excluding the 2009 season the same four plausible ICD-10 codes were identified. Restricting the analysis to only the 2009 influenza season, which was characterised by the pandemic, we found only three ICD-10 codes correlated with confirmed influenza cases. These correlations were also markedly strengthened (Table 5).

**Table 5:** Top correlations calculated between ICD-10 coded cases and NIDS cases during the influenza-seasons (n ≥ 200)

ICD-10 Code	Description	During influenza seasons. Not restricted		During influenza seasons & excluding 2009		2009 pandemic season	
		**Correlation**	**p**	**Correlation**	**p**	**Correlation**	**p**

B34	Viral infection, unspecified site	0.617	< 0.0001	0.575	< 0.0001	0.924	< 0.0001

J11	Influenza virus not identified	0.613	< 0.0001	0.545	< 0.0001	0.935	< 0.0001

J06	Acute upper respiratory infection multiple and unspecified sites	0.588	< 0.0001	0.525	< 0.0001	0.924	< 0.0001

R55	Syncope and collapse	*	*	*	*	0.634	0.0149

J22	Unspecified acute lower respiratory infection	0.355	< 0.0001	0.386	< 0.0001	0.523	0.1044

S06	Intracranial injury	0.310	< 0.0001	0.215	0.0050	*	*

T88	Complications of surgical or medical care not specified elsewhere	0.309	< 0.0001	*	*	*	*

J20	Acute bronchitis	0.270	0.0003	0.328	< 0.0001	0.116	0.6924

J00	Acute nasopharyngitis (common cold)	0.263	< 0.0004	0.203	0.0090	0.362	0.2036

J18	Pneumonia organism unspecified	0.253	0.0006	0.294	0.0001	0.384	0.1754

Z20	Contact & exposure to communicable disease	0.219	0.0030	*	*	*	*

T40	Poisoning by opium	0.211	0.0050	*	*	*	*

*Not in top 13 correlations or n < 200

There were 401 notified influenza cases during the non-influenza season (Note: the dataset did not contain records for the 2009 non-influenza season). Fifteen ICD-10 codes (13 codes after postcode restriction) had p values less than 0.05. The five largest correlations from each set are shown (Table 6). They were only weakly correlated with NIDS cases and none was a plausible surrogate for influenza like illness.

**Table 6:** Top correlations calculated between ICD-10 coded cases and NIDS cases in the non-influenza season

ICD-10 Code	Description	Not postcode restricted data (n ≥ 200 records)		Postcode restricted (n ≥ 200 records)	
		**Correlation**	**p**	**Correlation**	**p**

T40	Poisoning by opium	0.198	0.0020	0.173	0.0070

N30	Cystitis	0.194	0.0024	0.199	0.0017

F13	Mental/behavioral disorder due to sedatives hypnotics	0.192	0.0027		

A09	Infectious gastroenteritis and colitis, unspecified	0.186	0.0040		

S22	Fracture of ribs	0.175	0.0063		

L98	Pyogenic granuloma			0.181	0.0045

A41	Other sepsis			0.209	0.0011

A64	Unspecified sexually transmitted disease			0.164	0.0105

## Discussion

Syndromic surveillance has the potential to detect influenza outbreaks earlier than traditional methods [11,12]. In the event of a pandemic this could be of considerable benefit. However, it has not been clear which ICD-10 codes are actually used by emergency department staff when they see a patient with an influenza-like illness. It has also not been clear whether the same codes are used during the non-influenza season, nor the effect of a known pandemic on the number and distribution of each code in the emergency department data.

Our first observation was a marked increase in weekly counts of both laboratory-confirmed influenza cases in NIDS and relevant ICD-10 codes during the influenza pandemic period. The number of confirmed cases notified in 2009 was more than four times greater than the previous highest number reported, in 2007, even though the influenza-like-illness activity in the community was considered comparable to 2003 and 2007 [13]. This disparity between the notifications during a highly publicised pandemic period and previous seasons with similar influenza activity suggests that the true scale of a publicised outbreak cannot be accurately inferred by comparison with baseline data. The counts of the relevant ICD-10 codes were, however, not affected to the same extent.

Analysis of the data with the inclusion of the 2009 season identified five ICD-10 diagnoses that had moderate correlations with confirmed influenza cases: J11, J06, J22, B34, J18. The strongest correlations were found with three ICD-10 diagnosis codes: J11 (Influenza virus not identified), J06 (Acute upper respiratory infection multiple and unspecified sites) and J22 (Unspecified acute lower respiratory infection). These correlations were little altered by including lag periods. Restricting the data to postcodes of residence within a 10 km radius of the hospitals gave the same top five codes although with slightly smaller correlation coefficients, suggesting that the pattern of coding in these two urban hospitals closely follows the pattern of influenza across the whole state.

Excluding data from the 2009 influenza season resulted in no difference in the top ICD-10 codes identified for either the full dataset or postcode restricted data, although the correlations were slightly weaker. These 'best' five diagnosis codes were identified in an earlier study investigating correlations between ED presentations for influenza and notified influenza cases [10]. As in that study, combining the best five ICD-10 codes slightly strengthened the correlation over that found for J11 alone. The analysis of the 2009 influenza season only, found only three ICD-10 codes correlated with confirmed influenza cases: B34, J11 and J06. These results suggest that ED staff are much more likely to use certain codes when they know what is occurring in their catchment population. The best codes to use during the non-influenza season remains in doubt. The best five ICD-10 codes for the influenza season were not correlated with laboratory confirmed influenza notifications outside the influenza season, at least in part because of the small number of influenza cases notified during that period. There were no known 'out-of-season' outbreaks in our data set, but it seems unlikely that the single code of J11 would have been useful in detecting such an outbreak at a time that ED staff were not expecting to see influenza cases. We suggest using a combination of the 'best five' codes to track influenza outside the season, but a final conclusion awaits analysis of a data set that includes an 'out-of-season' outbreak.

## Conclusions

This study demonstrates that knowledge of circulating influenza virus in the community strongly influences the diagnostic codes used when patients present to emergency departments with influenza-like-illness. This has important implications for syndromic surveillance. The benefit of syndromic surveillance is to identify potential outbreaks when they are not expected, but the results of this study suggest that the ability of syndromic surveillance to detect outbreaks early may be impeded because case diagnosis is influenced by what ED staff believes to be occurring in the community.

## Competing interests

The authors declare that they have no competing interests.

## Authors' contributions

JB conceived the study, supervised the data analysis and edited the manuscript.

KM conducted the data analysis and drafted the manuscript. SR and LF assisted with data collection and editing of the manuscript. All of the authors read and approved the manuscript.

## Pre-publication history

The pre-publication history for this paper can be accessed here:

http://www.biomedcentral.com/1471-2458/11/338/prepub

## References

[B1] World Health Organization Influenza. Fact Sheet No.211 2009 http://www.who.int/mediacentre/factsheets/fs211/en/index.html

[B2] Department of Health and Ageing Australian Health Management Plan for Pandemic Influenza (2009) 2010 Canberra: Commonwealth of Australia http://www.flupandemic.gov.au/internet/panflu/publishing.nsf/Content/ahmppi-2009

[B3] Coory M Grant K Kelly H Influenza-like illness surveillance using a deputising medical service corresponds to surveillance from sentinel general practices Euro surveill 2009 14 pii = 19387 19941773

[B4] Hope K Durrheim D d'Espaignet E Dalton C Syndromic Surveillance: is it a useful tool for local outbreak detection? Journal of Epidemiology and Community Health 2006 60 374 5 10.1136/jech.2005.035337 16680907 PMC2563979

[B5] Elliot A Syndromic surveillance: the next phase of public health monitoring during the H1N1 influenza pandemic? Euro surveill 2009 14 pii = 19391 19941780

[B6] Buehler J Sonricker A Paladini M Syndromic surveillance practice in the United States: Findings from a survey of state, territorial and selected local health departments Biosecur Bioterror 2008 6 1 20

[B7] Griffin BA Jain AK Davies-Cole J Glymph C Lum G Washington SC Stoto MA Early detection of influenza outbreaks using the DC Department of Health's syndromic surveillance system BMC Public Health 2009 9 483 10.1186/1471-2458-9-483 20028535 PMC2807869

[B8] Lemay R Mawudeku A Shi Y Ruben M Achonu C Syndromic Surveillance for Influenzalike Illness Biosecurity and Bioterrorism: Biodefense Strategy, Practice, and Science 2008 6 161 70 10.1089/bsp.2007.0056 18563993

[B9] Marsden-Haug N Foster V Gould P Elbert E Wang H Pavlin J Code-based syndromic surveillance for influenzalike illness by International Classification of Diseases, ninth revision Emerg Infect Dis 2007 2 207 16 10.3201/eid1302.060557 PMC2725845 17479881

[B10] Newell M Black J Syndromic surveillance for influenza:how well do emergency department diagnoses correlate with notified cases Victorian Infectious Diseases Bulletin 2006 1 4

[B11] Brownstein J Mandl K Reeingineering real time outbreak detection systems for influenza epidemic monitoring AMIA Annual Symposium Proceedings 2006 866 PMC1839573 17238486

[B12] Zheng W Aitken R Muscatello D Churches T Potential for early warning of viral influenza activity in the community by monitoring clinical diagnoses of influenza in hospital emergency departments BMC Public Health 2007 7 250 10.1186/1471-2458-7-250 17877836 PMC2075512

[B13] Fielding J Higgins N Gregory J Grant K Catton M Bergeri I Lester R Kelly H Pandemic H1N1 influenza surveillance in Victoria, Australia, April - September, 2009 Euro surveill 2009 14 pii = 19368 10.2807/ese.14.42.19368-en 19883545

